# Marine Microalgae with Anti-Cancer Properties

**DOI:** 10.3390/md16050165

**Published:** 2018-05-15

**Authors:** Kevin A. Martínez Andrade, Chiara Lauritano, Giovanna Romano, Adrianna Ianora

**Affiliations:** Department of Integrative Marine Ecology, Stazione Zoologica Anton Dohrn, 80121 Naples, Italy; kevin.martinez@szn.it (K.A.M.A.); chiara.lauritano@szn.it (C.L.); romano@szn.it (G.R.)

**Keywords:** marine biotechnology, microalgae, anti-cancer

## Abstract

Cancer is the leading cause of death globally and finding new therapeutic agents for cancer treatment remains a major challenge in the pursuit for a cure. This paper presents an overview on microalgae with anti-cancer activities. Microalgae are eukaryotic unicellular plants that contribute up to 40% of global primary productivity. They are excellent sources of pigments, lipids, carotenoids, omega-3 fatty acids, polysaccharides, vitamins and other fine chemicals, and there is an increasing demand for their use as nutraceuticals and food supplements. Some microalgae are also reported as having anti-cancer activity. In this review, we report the microalgal species that have shown anti-cancer properties, the cancer cell lines affected by algae and the concentrations of compounds/extracts tested to induce arrest of cell growth. We also report the mediums used for growing microalgae that showed anti-cancer activity and compare the bioactivity of these microalgae with marine anticancer drugs already on the market and in phase III clinical trials. Finally, we discuss why some microalgae can be promising sources of anti-cancer compounds for future development.

## 1. Introduction

**Figure 1:**
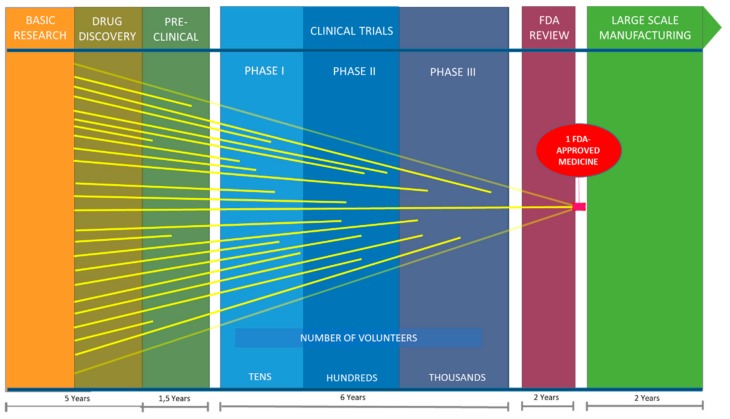
Time estimates for research and development of new Food and Drug Administration (FDA) approved drugs.

Cancer includes a large group of pathologies related to the unrestrained proliferation of cells in the body [1]. There are more than 200 different types of cancers, and some cancers may eventually spread into other tissues causing metastases that are often lethal. Cancer is the leading cause of death globally, largely due to aging and growth of the world’s population. According to the European Cancer Observatory [2], estimates for the four most common types of cancer in the European Union in 2012 were as follows: 342,137 cases of colon cancer, 309,589 cases of lung cancer (including trachea and bronchus cancer), 358,967 cases of breast cancer and 82,075 cases of skin melanoma. Finding more effective methods to treat cancer remains a challenge, and development of new therapeutic agents for cancer treatment is essential for continued progress against the disease. According to Dyshlovoy and Honecker [3] approximately 60% of the drugs used in hematology and oncology have their origin in natural sources, and one third of the most sold are either natural compounds or derivatives thereof. There has also been growing interest in marine bioprospecting, because potent natural compounds (e.g., terpenes, steroids, alkaloids, polyketides, etc.) have already been discovered from marine organisms. Currently there are seven drugs of marine origin on the market, four of which are anticancer drugs. There are also close to 26 marine natural products in clinical trials of which 23 are anti-cancer compounds [4]. Oceans cover nearly 70% of the planet, but remain largely unexplored. To date, more than 28,000 compounds isolated from marine organisms have been reported, and this number is rapidly growing each year [4]. However, despite the number of compounds isolated from marine organisms and the biological activities attributed to many of these, the search for ocean medicines is relatively recent and only in the middle part of the 20th century did scientists begin to systematically probe the oceans for new drugs. Today, the pipeline from the initial demonstration that a molecule may have therapeutic potential to the production of an approved drug involves pre-clinical testing, complex clinical trials in humans, and post-trial regulatory approval by the Food and Drug Administration (FDA). For drugs, this process can take 10 to 15 years (Figure 1) and costs millions of dollars [5], with less than 12% of the potential drugs receiving final approval [6].

Several factors, such as difficulties in harvesting organisms, low quantities of active compounds in extracts, finding adequate procedures for isolation and purification, possible toxicity of the compounds and sustainable production of compounds may slow down the entire pipeline. Notwithstanding these difficulties, the discovery of new ocean medicines is one of the most promising new directions of marine science today. Novel initiatives with marine organisms aimed at enabling environmentally-friendly approaches to drug discovery have been tackled in several European Union 7th Framework Programme (EU FP7) and European Union Horizon 2020 projects (EU H2020), such as Biologically Active Molecules of Marine Based Origin (BAMMBO), Bluegenics, European Marine Biological Research Infrastructure Cluster (EMBRIC), Genetic Improvement of Algae for Value Added Products (GIAVAP), Marine Microorganisms Cultivation Methods for Improving their Biotechnological Applications (MaCuMBA) and PharmaSea. This has led to several technological advancements in culturing micro- and macro-organisms, increased sampling efforts in diverse and often extreme habitats that have led to the discovery of species that are new for science, massive sequencing of genomes and transcriptomes allowing for the identification of new metabolic pathways and/or assignment of potential functions to unknown genes. Here, we discuss the marine microalgae which have shown anti-cancer activity. This is the first review on this subject because recent studies have indicated that microalgae may represent a reservoir for new bioactive compounds that can act as anti-cancer drugs.

## 2. Marine Microalgae

Microalgae are eukaryotic plants that contribute up to 40% of global productivity [7]. They are at the base of aquatic food webs, have short generation times (doubling time = 5–8 h for some species) and have colonized almost all biotopes, from temperate to extreme environments (e.g., cold environments and hydrothermal vents). Their advantage in marine drug discovery is their metabolic plasticity, which can trigger the production of several compounds with possible applications in various biotechnology sectors (i.e., food, energy, health, environment and biomaterials) [8,9]. They can be easily cultivated in photo-bioreactors (e.g., in 100,000 L bioreactors) to obtain a huge biomass and represent a renewable and still poorly-explored resource for drug discovery. They use solar energy and fix CO_2_ which contributes to the mitigation of greenhouse gas effects and the removal of nitrogen and phosphorous derivatives which can be pollutants depending on their concentration [10]. Table 1 reports the microalgal species that have shown anti-cancer properties, the cancer cell lines affected by microalgae and the concentrations that have been tested to induce the arrest of cell growth.

As reported in previous studies, the bioactivity of microalgae may differ for different clones and can vary depending on the culturing conditions (e.g., nutrient availability, temperature, light intensity) [8,27] and growth phase [28]. For example, Ingebrigtsen et al. [27] demonstrated that the bioactivity of various marine microalgae extracts (i.e., the diatoms *Attheya longicornis*, *Chaetoceros socialis*, *Chaetoceros furcellatus*, *Skeletonema marinoi* and *Porosira glacialis*) with anti-cancer activity against melanoma A2058 cells changed when they were cultured under different light and temperature conditions. Lauritano et al. [8] also showed that microalgal bioactivity can vary depending on the nutrient concentrations used for their cultivation. These authors showed that the diatom *Skeletonema marinoi* had anti-cancer activity exclusively when cultured under nitrogen starvation conditions. Considering the importance of culturing conditions (Table 2), we report the mediums used for growing marine microalgae that showed anti-cancer activity (e.g., Conway’s medium, Guillard’s F/2 medium or variations of both mediums) and, where available, the sampling locations and harvesting times.

## 3. Active Fractions from Marine Microalgae

### 3.1. Carotenoid Extract from Chlorella Ellipsoidea

*Chlorella* species are widely known as being a good commercial source of carotenoids such as lutein, β-carotene, zeaxanthin and astaxanthin. Kwang et al. 2008 [13] tested the anti-proliferative effect of the carotenoids extracted from the green algae *C. ellipsoidea* and *C. vulgaris* on a human colon carcinoma cell line (HCT116). Briefly, a freeze-dried *Chlorella* powder was extracted with ethanol, treated with a solution of KOH for saponification and further partitioned with hexane. This hexane phase, rich in carotenoids, was analyzed using HPLC-ESI-MS in order to identify the major carotenoid composition. They found that the carotenoid extract of *C. ellipsoidea* was mainly composed of violaxanthin and, in lower ratios, by two other xanthophylls (antheraxanthin and zeaxanthin). The extract from *C. vulgaris* was composed mainly of lutein. Anti-cancer activity was measured using the MTT assay after 24 h of exposure with the microalgal extracts. The half maximal inhibitory concentration (IC_50_) value was 40.73 ± 3.71 µg/mL for *C. ellipsoidea* and 40.31 ± 4.43 μg/mL for *C. vulgaris*—much higher than the IC_50_ of pure lutein (21.02 ± 0.85 µg/mL). In order to understand if apoptosis is linked to the observed anti-proliferative effect, the authors also performed an annexin V-fluorescein assay to check phosphatidylserine translocation (index of apoptosis). The apoptotic effect was confirmed after treatment with both *C. ellipsoidea* and *C. vulgaris* extracts. They reported that apoptosis was 2.5-fold higher in the case of the *C. ellipsoidea* extract. The carotenoid extract was not tested on normal human cell lines.

### 3.2. Ethanol and Ethyl Acetate Extracts from Chaetoceros Calcitrans

Nigjeh et al. [18] tested the ethanolic extract from the planktonic diatom *Chaetoceros calcitrans* on breast adenocarcinoma (MCF-7), breast epithelial (MCF-10A) and peripheral blood mononuclear cells (PMBC). The ethanolic extract was obtained after the homogenization of the microalgal biomass with absolute ethanol and further filtration of the supernatant using filter cotton and a 0.2 μm filtration unit. The results were compared with the effects of tamoxifen, an already known drug used for the treatment of breast cancer. Cell viability was performed using the MTT assay on MCF-7, MCF-10A and PMBC cells. The results were expressed as IC_50_ values obtained by screening different concentrations (0 to 30 µg/mL) of *C. calcitrans* extract for 24 and 72 h. The IC_50_ values of the ethanolic extract screened on MCF-7 were 3.00 ± 0.65 µg/mL for 24 h and 2.69 ± 0.24 µg/mL for 72 h, while the IC_50_ values of MCF-10A cells were 12.00 ± 0.59 µg/mL for 24 h and 3.30 ± 0.36 µg/mL for 72 h. *C. calcitrans* extract did not display any cytotoxicity on PMBC cells even at the highest concentrations; the activity was specific to the cancer cells. The IC_50_ of tamoxifen on MCF-7 was 12.00 ± 0.52 µg/mL for 24 h and 9.00 ± 0.40 µg/mL for 72 h. The comparison with tamoxifen shows that the anti-cancer activity of *C. calcitrans* extract is very interesting. In addition, annexin V/propidium iodide analyses were performed and the results indicated apoptosis induction in MCF-7 cells after treatment with the extract. The authors also observed an increase in the proapoptotic protein Bax, and the caspases 3 and 7 transcripts.

Goh et al. [19] analyzed the effects of the extracts from *C. calcitrans* against a wide range of cancer cell lines. In particular, they studied the cytotoxicity of four crude solvent extracts (hexane, dichloromethane, ethyl acetate and methanol) in the following cancer cell lines: human breast adenocarcinoma (MDA-MB-231), MCF-7, mouse breast carcinoma (4T1), liver hepatocellular carcinoma (HepG2), cervix epithelial carcinoma (HeLa), human prostate carcinoma (PC-3), human lung adenocarcinoma (A549), human colon adenocarcinoma (HT-29), and human ovarian adenocarcinoma (Caov3). A mouse embryo fibroblast (3T3) cell line was used to measure cytotoxicity against non-tumorigenic cells. A freeze-dried powder of *C. calcitrans* was shaken in hexane, dichloromethane, ethyl acetate or methanol (MeOH) for 24 h and filtered through cotton to obtain the different extracts. The MTT assay was carried out after 72 h of treatment with the microalgal extracts and doxorubicin was used as a control (60 µg/mL of doxorubicin). The authors observed that crude ethyl acetate extract from *C. calcitrans* had cytotoxic properties in the MDA-MB-231 cancer cell line with IC_50_ of 60 µg/mL. An assay on a non-tumorigenic fibroblast cell line also revealed that the cytotoxic effect was specific against cancer cells; the extract did not have cytotoxic effects on the 3T3 cell line.

### 3.3. Organic Fractions from Amphidinium Carterae

Samarakoon et al. [20] tested the anti-proliferative activity of various fractions from the dinoflagellate *Amphidinium carterae* extract on different cancer cell lines: HL-60 (Human promyelocytic leukemia cells), B16F10 (mouse melanoma tumor cells), and A549 (adenocarcinomic human alveolar basal epithelial cells). Cytotoxicity assays were also carried out using the mouse monocyte macrophage cell line (RAW 264.7). Freeze-dried biomass from the cultured marine microalgae was grounded into fine powder, extracted with methanol (80%) and homogenized by sonication at 25 °C for 90 min. The crude methanol extract was concentrated by evaporating the solvent under reduced pressure using a rotary evaporator and further partitioned. Analytical grade n-hexane, chloroform, ethyl acetate, and water were used in solvent-solvent partition chromatography in order to obtain the fractions to be tested. Cell growth inhibition was measured with the MTT assay. *A. carterae* chloroform fraction was the most active and reduced HL-60 cell viability by about 50% after 24 h exposure at a concentration of 50 µg/mL. No tests were performed on normal human cell lines.

### 3.4. Methanolic Extracts from Amphidinium Carterae, Prorocentrum Rhathymum, Symbiodinium sp., Coolia Malayensis, Ostreopsis Ovata, Amphidinium operculatum and Heterocapsa psammophila

Shah et al. [21] cultivated up to eleven different strains of benthic dinoflagellates (*Amphidinium carterae*, *Prorocentrum rhathymum*, *Symbiodinium sp.*, *Coolia malayensis* strain 1, *Ostreopsis ovata* strain 1, *Ostreopsis ovata* strain 2, *Coolia malayensis* strain 2, *Amphidinium operculatum* strain 1, *Heterocapsa psammophila*, *Coolia malayensis* strain 3 and *Amphidinium operculatum* strain 2) isolated from the coast of Jeju Island (Korea) in 2011, to screen on RAW 264.7 (murine macrophage cell line) and HL-60 (human promyelocytic leukemia cell line) cells. They specified the specific sampling location and the microalgal growth phase tested (Table 2). To obtain the methanolic extracts, the freeze-dried biomass from the cultured marine microalgae was ground into fine powder, extracted with methanol (80%) and homogenized by sonication at 25 °C for 90 min. An MTT assay was carried out to study cell viability after extract exposure for 24 h at 37 °C. In this case, only *Ostreopsis ovata* 1 and *Amphidinium operculatum* 1 significantly inhibited the growth of HL-60 cancer cells (reducing cell viability between 40 and 60% compared to the control, at a concentration of 50 µg/mL). No tests were performed on normal human cell lines.

### 3.5. Hydrophobic Fraction from Skeletonema Marinoi

Lauritano et al. [8] studied the effects of 32 species of microalgae identified by microscopy and 18S sequencing. The microalgal biomass from the cultured marine microalgae was extracted with a ratio of acetone:water (1:1) and further fractionated using Amberlite RXAD16N resin with acetone as the resin eluent to obtain an hydrophobic fraction. The authors found that hydrophobic fractions from *Skeletonema marinoi*, *Alexandrium minutum*, *Alexandrium tamutum* and *Alexandrium andersoni* were active against a melanoma cancer cell line (A2058) at a concentration of 100 μg/mL. Further testing on a normal lung fibroblast (MRC-5) cell line showed that *Alexandrium* species were toxic. Two different strains of *Skeletonema marinoi* (FE6 and FE60 strains from the Adriatic Sea) were tested on an A2058 cell line. The colorimetric MTS (3-(4,5-dimethylthiazol-2-yl)-5-(3-carboxymethoxyphenyl)-2-(4-sulfophenyl)-2*H*-tetrazolium, a tetrazolium dye used for the quantification of viable cells) assay was performed to check the cytotoxicity in both normal and cancer cell lines. The results showed that only the FE60 strain was active against A2058 and only when cultured under nitrogen-starvation conditions. FE60 reduced the cell viability to 60% when screened at 50 µg/mL and to 10% when screened at 100 µg/mL.

### 3.6. Aqueous Extract from a Canadian Marine Microalgal Pool

Somasekharan et al. [25] studied the anti-proliferative effect of a raw marine microalgal material from Canada (dried powder) on eight different cancer cell lines, together with the anti-colony forming activity in the same lines. The dried microalgal powder was suspended in distilled water at a concentration of 30 mg/mL and then sonicated with short bursts. The sonicated suspension was then passed through a 25-gauge needle to release the cytosolic contents, followed by syringe filtration through 0.2 μm filters. This aqueous extract from the raw material was tested on A549, H460 (lung adenocarcinoma cell lines), PC-3, DU145 (prostate cancer cell lines), N87 (stomach cancer cell line), MCF7 (breast cancer cell line), BxPC-3 (pancreas cancer cell line) and MNNG (bone cancer cell line). The cells were incubated with the extract for 72 h at 0 (control), 1, 2 and 5 mg/mL. Cell viability was determined using the MTT assay. The extract did not show any significant activity at 1–2 mg/mL except for on the MNNG cell line (50% reduction in cell viability at 2 mg/mL). At 5 mg/mL proliferation of almost all the cell lines were significantly inhibited. Tests on normal human cell lines were not performed. The authors performed a crystal violet test at 0.5–5 mg/mL to check the anti-colony activity of the extract. This test indicated that the extract successfully inhibited the colony forming ability of all cancer cells tested even at the lowest concentration (0.5 mg/mL).

### 3.7. Aqueous Extract from Chlorella Sorokiniana

*Chlorella* sp. biomass is widely used as a dietary supplement in many countries and is mostly produced in Asia (https://www.marketresearchfuture.com/reports/chlorella-market-4413). There are also some patents related to its use as a dietary supplement (e.g., US 2005/0196389 A1 [29]). Lin et al. [26] studied the effects of hot water extracts from the diatom *Chlorella sorokiniana* (marine strain) on lung adenocarcinoma cell lines (A549 and CL1-5). The extracts were obtained by reflux extraction of the dried biomass with distilled water for 1 h and further filtration with N0.5 filter paper. To determine the cytotoxicity, the authors performed the MTT assay at a concentration range of 15.625 to 1000 ng/mL; the results indicated a dose-dependent reduction in cell viability on both cancer cell lines. The cytotoxicity on normal human cells was not tested. They also studied the mechanism of action of *C. sorokiniana* extract using annexin V/propidium iodide staining (flow cytometry analysis) to confirm a possible cell cycle arrest/apoptotic process. Cell cycle arrest was not observed even after 24 h of exposure, but an increment in the number of cells in sub-G1 phase was observed, which is a phenomenon that typically indicates apoptosis. Protein expression (Western blot analysis) of the cleaved and activated forms of caspase 9, caspase 3 and Poly (ADP-ribose) polymerase (PARP) increased in both cell lines after exposure to the microalgal extract after 24 h. The activation of caspase 9 and caspase 3 suggested that the main pathway involved in apoptosis was the mitochondrial pathway. In addition, the ratio of Bax/Bcl-2 (pro/antiapoptotic proteins) increased after 24 h of treatment which is another sign of apoptosis.

## 4. Active Compounds from Marine Microalgae

### 4.1. Polyunsaturated Aldehides (PUAs)

**Figure 2:**
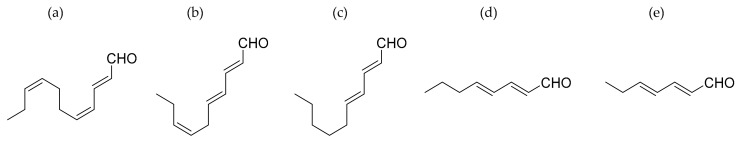
Polyunsaturated aldehydes. From left to right: 2-*trans*-4-*cis*-7-*cis*-decatrienal (**a**); 2-*trans*-4-*trans*-7-*cis*-decatrienal (**b**); 2-*trans*-4-*trans*-decadienal (**c**); 2-*trans*-4-*trans*-octadienal (**d**) and 2-*trans*-4-*trans*-heptadienal (**e**).

Miralto et al. [11] isolated three polyunsaturated aldehydes (PUAs, Figure 2) from the marine diatoms *Thalassiosira rotula*, *S. costatum* and *P. delicatissima*. They found that 2-*trans*-4-*cis*-7-*cis*-decatrienal, 2-*trans*-4-*trans*-7-*cis*-decatrienal and 2-*trans*-4-*trans*-decadienal had anti-proliferative activity on the human colon adenocarcinoma cell line (Caco-2). To check the anti-proliferative activity, they used different concentrations of PUAs between 0 and 20 µg/mL after 48 h of incubation. Concentrations of 11–17 µg/mL were enough to reduced cell viability to almost 0%. In addition, a TUNEL assay was performed to check DNA fragmentation and to verify that apoptosis had occurred. 

Sansone et al. [12] tested the effect of the commercially-available PUAs 2-*trans*-4-*trans*-decadienal (DD), 2-*trans*-4-*trans*-octadienal (OD) and 2-*trans*-4-*trans*-heptadienal (HD) (Figure 2) on the adenocarcinoma cell lines, lung A549 and colon COLO 205. The authors tested these three polyunsaturated aldehydes at different exposure times (i.e., 48 and 72 h) and concentrations (i.e., 2, 5 and 10 µM). For quantities of 2, 5 and 10 µM DD decreases in cell viability of 70%, 50% and 18% respectively, were induced in A549 cells after 24 h. For the COLO 205 cell line, cell viability decreased to 80%, 44% and 26% with 2, 5 and 10 µM DD, respectively. In the case of OD, 10 µM of the compound decreased cell viability to 35% after 72 h in A549 cells. At 2, 5 and 10 µM, OD also reduced cell viability to 60%, 60% and 41% in COLO 205 cells, respectively, after 72 h. At 10 µM, HD reduced cell viability to 10% in A549 cells after 48 h and 0% after 72 h, while the same concentration tested on COLO 205 cells reduced cell viability to 40% after 48 h and 28% after 72 h. The authors also tested the three PUAs on a normal lung/brunch epithelial BEAS-2B cell line to check cytotoxicity on normal cells. None of the PUAs were toxic.

### 4.2. Chrysolaminaran Polysaccharide

**Figure 3:**
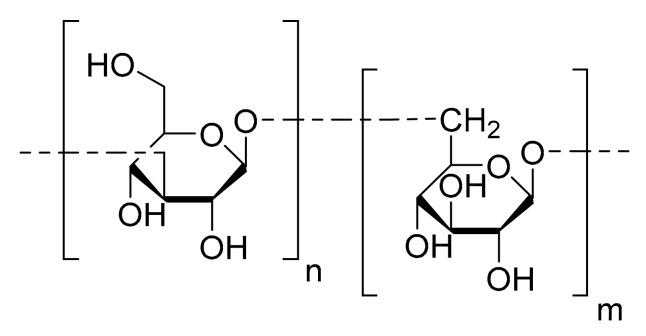
Chrysolaminaran monomer.

Kusaikin et al. [14] isolated one polysaccharide of the chrysolaminaran family (Figure 3) from the diatom *Synedra acus*. These storage polysaccharides are well known to be common water soluble biopolymers synthetized by diatoms [30]. The anti-tumor activity of the chrysolaminaran extracted from *S. acus* was studied on HTC-116 and DLD-1 human colon cancer cell lines. The MTS method was carried out to determine cell viability. Cancer cells were treated with 25, 50 and 100 µg/mL of chrysolaminaran for up to 72 h. The inhibition trend in the different experiments was irregular, but the IC_50_ values were determined for each cell line: 54.5 µg/mL for HCT-116 and 47.7 µg/mL for DLD-1. The authors did not find any toxicity on the HTC-116 and DLD-1 cell lines at concentrations above 200 mg/mL and, considering that most anti-tumor drugs are toxic at these concentrations, this is a very promising property. The degree of cytotoxicity on normal human cells was not tested.

### 4.3. Violaxanthin

**Figure 4:**
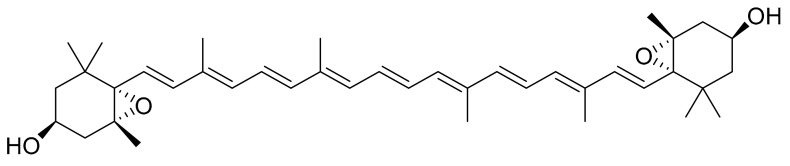
Violaxanthin.

Pasquet et al. [15] performed an anti-cancer screening of extracts from the green algae *Dunaliella tertiolecta* on four different cancer cell lines: MCF-7, MDA-MB-231, A549 and LNCaP. They prepared different extracts using a wide range of solvents in terms of polarity, including dichloromethane, ethanol and ultrapure water. The MTT assay was used to evaluate the cells’ viability. The dichloromethane extract showed significant activity against MCF-7 cancer cells. RP-HPLC analysis and fractionation was used to obtain one subfraction of the dichloromethane extract that was also screened for 72 h at concentrations between 0.1 µg/mL and 40 µg/mL. The subfraction was then identified as violaxanthin (Figure 4) at a rate of 95%. In addition to these results, the DNA of non-treated and treated cells was extracted and analysed using standard electrophoresis. Despite indications of early apoptosis (phosphatidylserines translocation detected using annexin-V-Alexa 568 fluorochrome), the violaxanthin subfraction did not cause any DNA fragmentation. Cytotoxicity tests were not performed in normal human cell lines.

### 4.4. Eicosapentaenoic Acid (EPA)

**Figure 5:**
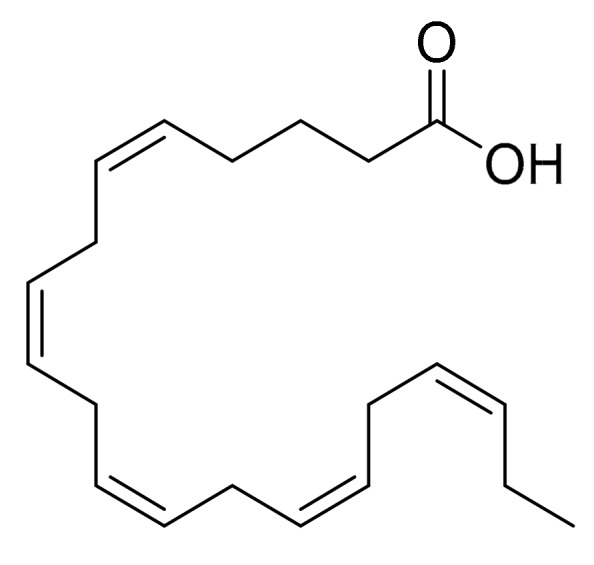
Eicosapentaenoic acid.

Nappo et al. [16] screened the extracts from the marine diatom *Cocconeis scutellum* on the following cells lines: BT20 (human breast cancer), MB-MDA468 (human breast cancer), LNCaP (human prostate adenocarcinoma cells), COR (Epstein-Barr Virus-transformed B cells isolated from human tonsils), JVM2 (lymphoblast immortalized with Epstein-Barr virus) and BRG-M (Burkitt’s lymphoma cells). The results of the screening were not entirely published but the authors determined that *C. scutellum* extract was more effective on the BT20 cell line. The degree of cytotoxicity normal human cell lines was not studied. The fractionation of diethyl ether extract (the most active) from *C. scutellum* produced three fractions with differentiated activities. Fractions 1–2 did not induce any significant reduction in cell viability compared to the control but fraction 3 reduced the viability to 56.2%. DNA fragmentation was evaluated with the annexin V/propidium iodide staining methods. The analysis of the composition of these fractions indicated that fraction 1 contained glycerides (77.2% of total ion current) and fatty acids (2.4%), fraction 2 contained fatty acids (66.7%), monoglycerides (11.0%) and sterols (3.2%), and finally, fraction 3 contained fatty acids (81.7%) and 4-methylcholesterol (2.3%). The authors concluded that the fatty acid subfractions were responsible for this activity, specifically, eicosapentaenoic acid (EPA, Figure 5). This conclusion was reached because EPA was the only product in the fraction that has been reported to induce apoptosis [31]. The activation of caspases 8 and 3 was also confirmed by Western blot analysis. The authors concluded that it is not yet clear whether EPA is the only factor involved in the apoptosis of BT20 cells or if there is a synergic association among different compounds in the same fraction.

### 4.5. Fucoxanthin

**Figure 6:**
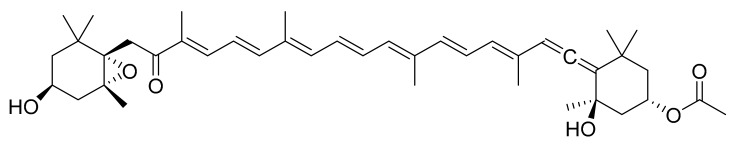
Fucoxanthin.

Fucoxanthin (Figure 6) is one of the most studied compounds that can be found in marine micro- and macroalgae. It is a pigment from the family of the xanthophylls and a major carotenoid in brown algae [32]. Kadekaru et al. [33] evaluated fucoxanthin toxicity by providing oral doses (10 mg/kg and 50 mg/kg) to rats for a period of 28 days. Fucoxanthin did not show any obvious toxicity and is hence considered safe as a pharmaceutical ingredient. Ishikawa et al. [34] performed a similar analysis in mice using a metabolite of fucoxanthin, fucoxanthinol. In this case, they used a higher dose (200 mg/kg) for 28 days, but it did not show any toxicity either.

Hosokawa et al. [35] showed that fucoxanthin had strong anti-proliferative activity against HL-60 cells and could also induce apoptosis. Cells treated with 11.3 and 45.2 µM of fucoxanthin showed viabilities of 46.0% and 17.3%, respectively, after 24 h. Cell viability was determined by the dye exclusion test using trypan blue. Hosokawa et al. 2004 also tested the cell viability on three human colon cancer cell lines (Caco-2, DLD-1 and HT-29) treated with fucoxanthin. Caco-2, DLD-1 and HT-29 cells showed a dose-time dependent trend. The Caco-2 cell line was more affected than the other two cell lines (analyzed by WST-1 assay). Normal human cells were not tested.

Kotake-Nara et al. [36] examined 15 types of carotenoids (Neoxanthin, fucoxanthin, phytofluene, lycopene, phytoene, canthaxanthin, β-cryptoxanthin, zeaxanthin, β-carotene, α-carotene, γ-carotene, astaxanthin, capsanthin, lutein and violaxanthin) on three different prostate cancer cell lines (PC-3, DU145 and LNCaP) and found that fucoxanthin was one of the most active anti-cancer compounds. The percentages of viable cells after 72 h when fucoxanthin was added at 20 µM were 14.9% for PC-3, 5.0% for DU145 and 9.8% for LNCaP, respectively (determined by MTT assay). Normal human cells were not tested.

Peng et al. [17] summarized the studies related to fucoxanthin, the microalgae that are known to produce it (i.e., *Chaetoseros* sp., *Cylinrotheca closterium*, *Odontella aurita* and *Phaeodactylum tricornutum*) and its role as a bioactive compound (e.g., as an antioxidant, anti-inflamatory, anti-cancer, anti-diabetic, skin protective agent, bone protective agent, etc.).

### 4.6. Stigmasterol

**Figure 7:**
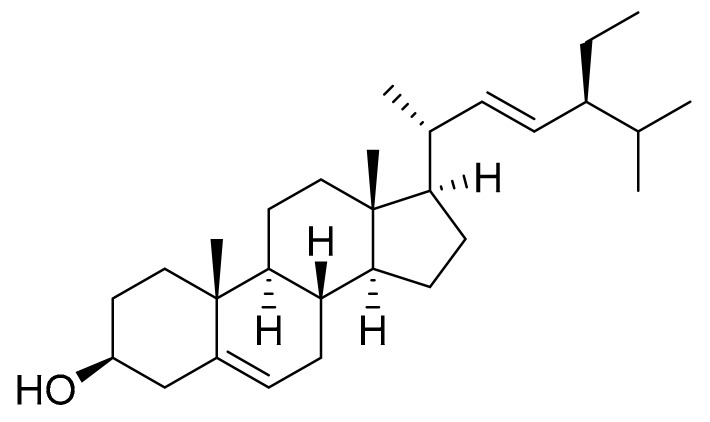
Stigmasterol.

Kim et al. [22] isolated Stigmasterol (Figure 7) from *Navicula incerta* extracts using chromatography techniques such as silica gel open column chromatography and preparative thin layer chromatography (PTLC). They screened the anti-proliferative effect of the isolated stigmasterol at 5, 10 and 20 μM on HepG2 (human liver cancer cell line). Cytotoxicity values of 40%, 43% and 54% were found, respectively, which indicated a dose-dependent trend. The cytotoxic effects on normal human cells were not studied. The phytosterol-like structures with double bonds in the C-5 and C-22 positions, like stigmasterol, have been shown to induce apoptosis [37]. In this case, apoptosis was studied by controlling morphological changes, fluorescence-activated cell sorting, apoptosis pathways analysis, gene expression levels and also, with flow cytometric measurement of cell cycle arrest. All these assays indicated that stigmasterol has a huge apoptosis induction capability, probably via an apoptosis signaling pathway in the mitochondria.

### 4.7. NAMO (Nonyl 8-acetoxy-6-methyloctanoate)

**Figure 8:**
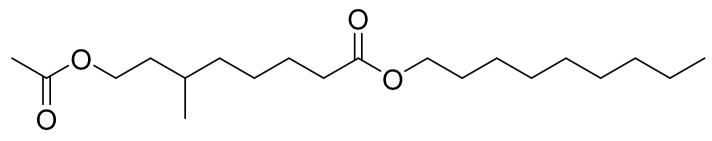
Nonyl 8-acetoxy-6-methyloctanoate (NAMO).

Samarakoon et al. [23] tested the anti-cancer activity of 8-acetoxy-6-methyloctanoate (NAMO, Figure 8) obtained from *Phaeodactylum tricornutum* against three different cell lines: human promyelocytic leukemia cell line (HL-60), a human lung carcinoma cell line (A549) and a mouse melanoma cell line (B16F10). NAMO was screened at 25 and 50 µg/mL for 48 h. NAMO was only active against HL-60 cells at both concentrations tested. The highest growth inhibitory activity of about 70% on HL-60 cells was observed at a concentration of 50 µg/mL NAMO. The cytotoxic effects on normal human cells were not studied. Regarding its mechanism of action, NAMO induced DNA damage and increased apoptotic body formation. Cell cycle arrest and the accumulation of cells in the sub-G1 phase were observed to occur proportionally to the concentration of NAMO. The authors also observed activation of the pro-apoptotic protein Bax, suppression of the anti-apoptotic protein Bcl-x, and an increase in the expression of both caspase-3 and p53 proteins.

### 4.8. Monogalactosyl Glycerols

**Figure 9:**
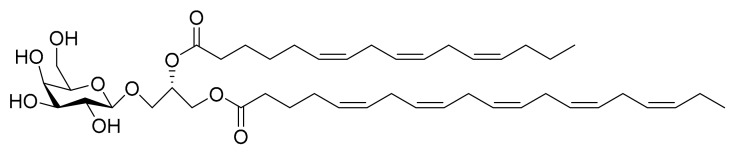
Monogalactosyl Glycerol (Compound **1**): (2*S*)-1-*O*-5,8,11,14,17-eicosapentaenoyl-2-*O*-6,9,12-hexadecatrienoyl-3-*O*-[β-d-galactopyranosyl]-glycerol.

**Figure 10:**
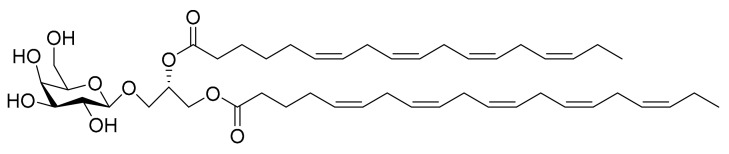
Monogalactosyl Glycerol (Compound **2**): (2*S*)-1-*O*-3,6,9,12,15-octadecapentaenoyl-2-*O*-6,9,12,15-octadecatetraenoyl-3-*O*-β-d-galactopyranosyl-sn-glycerol.

Andrianasolo et al. [24] isolated two different monogalactosyl glycerols (Figure 9 and Figure 10) from *Phaeodactylum tricornutum* and tested them against immortal mouse epithelial cells (W2 and D3). The W2 cell line is a wild type, while D3 cells have the apoptosis function disabled through gene deletion (this assay is one of the approaches for the study of apoptosis and its role in cancer and oncogenesis). The minimum values required for apoptosis induction with this test were a death rate of 20% on the W2 cell line and, at a growth rate of at least 10% on the D3 cell line. For compound **1** (Figure 9) (52 µM) the W2 death rate was 18% ± 1% and the D3 growth rate was 10% ± 1%. For compound **2** (Figure 10) (64 µM), the W2 death rate was 18% ± 1% and the D3 growth rate was 14% ± 1%. The results confirmed that the isolated compounds have specific apoptosis activity against the W2 cell line.

## 5. Active Compounds from Other Marine Organisms

To better understand the potential of microalgal species as important sources of anti-cancer compounds, we compared their bioactivity with marine anti-cancer drugs already on the market. Table 3 and Table 4 report the anti-cancer compounds already on the market and in phase III clinical trials, the marine organisms from which the compounds were isolated, the active compounds, the target cancer cell lines and the active concentrations that arrest the growth of cancer cells.

These studies give an overview on the active concentrations for each compound and delimit a concentration range (0.365–272 ng/mL IC_50_) that can be used to understand whether a compound is active enough to be considered as a drug candidate. The sources of the active compounds in all of these cases were multicellular organisms which implies that there are difficulties in harvesting biomass and obtaining low amounts of secondary metabolites and a huge environmental impact due to incorrect harvesting [4]. Most of the compounds in Table 3 and Table 4 are small-medium size molecules, but of particular interest is brentuximab vedotin, an antibody-drug conjugate where the compound monomethyl auristatin E is linked to a monoclonal antibody (mAb) that recognizes a specific marker expression in cancer cells and directs monomethyl auristatin E to the targeted cancer cell. The reason why it is used as a complex and not as a pure compound is because it is too potent (100–1000 times more potent than doxorubicin, a common drug used for chemotherapy) and less specific to cancer cells [45].

## 6. Discussion

Anti-cancer compounds from marine microalgae have been poorly investigated. Most studies have been done on microalgal extracts or fractions obtained using low resolution methods such as liquid-liquid partitioning or solid phase extractions. It is uncommon to see dereplication methodologies, fractionations based on high throughput techniques (e.g., HPLC or gas chromatography) or a complete structural elucidation of the compounds that have been found.

Despite the low availability of data so far, the studies performed on *Chlorella sorokiniana* and *Chaetoceros calcitrans* show interesting activities compared to commercially available marine anti-cancer drugs [18,26]. Most of the marine pharmaceuticals on the market are active at the level of 0.6–7 ng/mL (Table 3) while the fractions from *Chlorella sorokiniana* and *Chaetoceros calcitrans* display significant activity at 1–3 µg/mL (Table 1). Even if fractions and pure compounds cannot be directly compared in terms of activity, the anti-cancer activities of *C. sorokiniana* and *C. calcitrans* extracts seem very promising and appear preferential for further investigation and purification of the active molecules. Considering that 0.0001% of the crude aqueous ethanol extract of the ascidian *Ecteinascidia turbinate* [46] leads to the isolation of trabectedin ET-743 and the development of the anti-cancer drug Yondelis, it would not be difficult to find a compound from *C. sorokiniana* and *C. calcitrans* with an activity as high as that of the marketed drugs. These data highlight that microalgae can be a promising source of anti-cancer compounds. Regarding the other fractions/extracts obtained from marine microalgae, it cannot be excluded that other compounds are present with clinical or biotechnological potential, but further studies are necessary to demonstrate this possibility.

Several compounds isolated from microalgae mentioned in this review have been studied not only as possible anti-cancer compounds, but also for other biotechnological applications. Compounds such as polyunsaturated aldehydes [11,12], eicosapentaenoic acid (EPA) [16], fucoxanthin [17], violaxanthin [15], stigmasterol [22] or chrysolaminaran [14] (Table 1) may have a potential role as high-value products (e.g., nutraceuticals, cosmeceuticals, additives, fuel precursors and biomaterials) or as possible future new drugs. For example, the polyunsaturated aldehydes have shown anti-proliferative activities on Caco-2 [11], A549 and COLO 205 cancer cell lines [12], but also anti-bacterial activities [47,48], making them possible new candidates for drug development. The polyunsaturated fatty acid, EPA, has been widely studied as a nutraceutical or dietary supplement, with beneficial effects on fetal development, prevention of cardiovascular diseases and even, the improvement of cognitive functioning in patients with Alzheimer’s disease [49].

The carotenoids, fucoxanthin and violaxanthin, have been used as precursors of vitamins in food and animal feed, additives, cosmetics, food coloring agents and biomaterials [50]. In particular, fucoxanthin has been shown to possess potential anti-inflammatory, antioxidant, anti-obesity, anti-diabetic, anti-tumorigenic and cardioprotective activities [51]. On the market, there are several fucoxanthin-based products used as dietary supplements such as “Solaray Fucoxanthin Special Formula Vegetarian Capsules” or “BRI NUTRITION ^®^ Fucoxanthin Capsules”.

Phytosterols such as stigmasterol have been receiving increasing attention because of their capacity to reduce blood cholesterol concentrations, prevent cardiovascular disorders and because of their health benefits in general [52]. Stigmasterol has been studied not only for its anti-cancer activity [22] but also for its antioxidant activity [53]. On the market, the phytosterol, β-sitosterol, is sold as a nutraceutical under brand names like “NOW ^®^ Foods Beta-Sitosterol Plant Sterols Softgels”. β-sitosterol has been widely studied for several biological activities such as anti-inflammatory, antioxidant, anti-diabetic or anti-cancer activities [54].

Finally, microalgal polysaccharides have the capacity to modulate the immune system and inflammatory reactions, making them interesting candidates for cosmetic additives, food ingredients and natural therapeutic agents [50]. Chrysolaminaran, for example, is a storage polysaccharide that is well known to be a common water soluble biopolymer synthetized by diatoms. Water soluble storage carbohydrates are more accessible, energetically and biophysically, than insoluble starch [30] so they can also be useful for microalgal energy applications. 

There is sufficient evidence confirming the key roles of culturing conditions (e.g., light intensity, photoperiod, nutrient availability or harvesting time) in the modification of microalgal bioactivity. Studies such as those by Lauritano et al. [8], Ingebrigten et al. [27] and Ribalet et al. [28] have shown how light intensity, temperature, nutrient concentration and harvesting time have strong impacts on the activity of different microalgal strains. The use of stressful conditions (such as nutrient starvation, light or temperature variation) seems to play a key role in the production of active metabolites. Unfortunately, very few studies on anti-cancer activities from microalgae have reported the culturing conditions and the growth phases at which the species were tested in detail (Table 2). This makes stressful culturing conditions still an unexploited tool with a high potential for the bioprospecting of novel bioactive metabolites from marine microalgae.

## Figures and Tables

**Table 1:** Active microalgal species, active fraction/compounds tested and cell lines against which these have proven to be effective (CV stands for cell viability).

Microalgae	Fraction/Compound	Target Cells	Active Concentration	Reference
*Thalassiosira rotula, Skeletonema costatum and Pseudonitzschia delicatissima.* Commercial source, not from microalgae	Polyunsaturated aldehydes (PUAs)	Colon adenocarcinoma (Caco-2) Lung adenocarcinoma (A549) Colon adenocarcinoma (COLO 205)	11 to 17 µg/mL (arrest of cell growth) 0.22 to 1.5 µg/mL (CV of 80% to 0% depending on the conditions)	[11] [12]
*Chlorella ellipsoidea*	Carotenoid extract	Colon carcinoma (HCT-116)	40 µg/mL (IC_50_)	[13]
*Synedra acus*	Chrysolaminaran (polysaccharide)	Colorectal adenocarcinoma (HT-29 and DLD-1)	54.5 and 47.7 µg/mL (IC_50_ for HT-29 and DLD-1)	[14]
*Dunaliella tertiolecta*	Violaxanthin (carotenoid already identified in *C. ellipsoidea*)	Breast adenocarcinoma (MCF-7)	40 µg/mL (to observe cytostatic activity)	[15]
*Cocconeis scutellum*	Eicosapentaenoic acid (EPA)	Breast carcinoma (BT20)	Not clarified	[16]
*Chaetoseros sp., Cylinrotheca closterium, Odontella aurita* and *Phaeodactylum tricornutum*	Fucoxanthin (carotenoid)	Promyelocytic leukemia (HL-60), Caco-2, colon adenocarcinoma (HT-29), DLD-1 and prostate cancer (PC-3, DU145 and LNCaP)	29.78 µg/mL (CV of 17.3% for HL-60) 10.01 µg/mL (CV of 14.8%, 29.4% and 50.8% for Caco-2, DLD-1 and HT-29) 13.18 µg/mL (CV of 14.9%, 5.0% and 9.8% for PC-3, DU145 and LNCaP)	[17]
*Chaetoceros calcitrans*	EtOH extract AcOEt extract	MCF-7 Breast adenocarcinoma (MDA-MB-231)	3.00 µg/mL (IC_50_) 60 µg/mL (IC_50_)	[18] [19]
*Amphidinium carterae*	CH_3_Cl fraction Hexane fraction AcOEt fraction	HL-60 HL60, Skin melanoma (B16F10), A549	50 µg/mL (CV of 40%) 25–50 µg/mL (CV between 50% and 90%)	[20]
Eleven strains of benthic diatoms *Ostreopsis ovata* *Amphidinium operculatum*	MeOH extract	HL-60	50 µg/mL (CV of 48% for *O. ovata* and 58% for *A. operculatumi*)	[21]
*Navicula incerta*	Stigmasterol (phytosterol)	Liver hepatocellular carcinoma (HepG2)	8.25 μg/mL (CV of 54%)	[22]
*Phaeodactylum tricornutum*	Nonyl-8-acetoxy-6-methyloctanoate (NAMO, fatty alcohol ester) Monogalactosyl glycerols ^1^	HL-60 Mouse epithelial cell lines (W2, D3)	22.3 μg/mL (IC_50_) 40-50 μg/mL (concentration necessary to induce apoptosis)	[23] [24]
*Skeletonema costatum* *Skeletonema marinoi*	Hydrophobic fraction and PUAs Hydrophobic fraction	Caco-2 (A2058 not affected) Skin melanoma (A2058)	11 to 17 µg/mL (PUAs) 50 µg/mL (CV of 60%)	[11] [8]
*Canadian marine microalgal pool*	Aqueous extract	A549, lung carcinoma (H460), prostate carcinoma (PC-3, DU145), stomach carcinoma (N87), MCF-7, pancreas adenocarcinoma (BxPC-3) and osteosarcoma (MNNG)	5000 µg/mL (CV between 30% and 80% depending on the cell line)	[25]
*Chlorella sorokiniana*	Aqueous extract	A549 and lung adenocarcinoma (CL1-5)	0.0156 to 1 µg/mL (CV reduced down to 20% progressively)	[26]

^1^ (2*S*)-1-*O*-5,8,11,14,17-eicosapentaenoyl-2-*O*-6,9,12-hexadecatrienoyl-3-*O*-[β-d-galactopyranosyl]-glycerol and (2*S*)-1-*O*-3,6,9,12,15-octadecapentaenoyl-2-*O*-6,9,12,15-octadecatetraenoyl-3-*O*-β-d-galactopyranosyl-sn-glycerol.

**Table 2:** Active microalgal species, sources, culturing conditions and references.

Microalgae	Source	Culturing Conditions	Harvesting Time	Reference
*Synedra acus*	Lake Baikal	Culture medium consisting of (mg/L) Ca(NO_3_)_2_·4H_2_O (20), KH_2_PO_4_ (2), MgSO_4_ (12), NaHCO_3_ (30), Na_2_EDTA (2.2), H_3_BO_3_ (2.4), MnCl_2_·4H_2_O (1.3), (NH_4_)_6_Mo_7_O_24_·4H_2_O (1), Na_2_SiO_3_·9H_2_O (25), FeCl_3_ (1.6), cyanocobalamine (0.04), thiamine (0.04), and biotin (0.04). 12 °C and 250–300 µmol·m^−2^·s^−1^ light intensity.	Not provided	[14]
*Dunaliella tertiolecta*	DT strain CCMP364 (NCMA, USA)	Conway medium. 20 °C, 180 μmol·m^−2^·s^−1^ light intensity.	Late exponential phase	[15]
*Cocconeis scutellum*	Mediterranean Sea, Stazione Zoologica A. Dohrn	Guillard’s F/2 medium.18 °C, 140 µmol·m^−2^·s^−1^ light intensity and 12 h:12 h photoperiod.	Not provided	[16]
*Chaetoceros calcitrans*	Strain UPMAAHU10 University Putra Malaysi	Conway medium. 24 °C, 120 μmol·m^−2^·s^−1^ light intensity, automatic oscillating shaker at 110 rpm and harvested at stationary phase (6–7 days). Conway medium. Conditions not provided.	Stationary phase Not provided	[18] [19]
*Amphidinium carterae*	Korea Marine Microalgae Culture Center	Conway medium. 20 °C, 34 μmol·m^−2^·s^−1^ light intensity and 24 h:0 h photoperiod.	Days 8–10	[20]
Eleven strains of benthic dinoflagellates	Coast of Jeju Island (Korea)	Daigo IMK medium (Nihon Pharmaceutical Co., Ltd.) and Guillard’s F/2 medium. 20 °C, 180 μmol·m^−2^·s^−1^ light intensity and 12 h:12 h photoperiod.	Exponential phase.	[21]
*Navicula incerta*	Korea Marine Microalgae Culture Center.	Guillard’s F/2 medium. Conditions not provided.	Not provided	[22]
*Phaeodactylum tricornutum*	Korea Marine Microalgae Culture Center Provasoli-Guillard National Center	Conway medium. 20 °C, 34 μmol·m^−2^·s^−1^ light intensity and 24 h:0 h photoperiod. Guillard’s F/2 medium. 18 °C and 100 μmol·m^−2^·s^−1^ light intensity.	Days 8–10 Not provided	[23] [24]
*Skeletonema marinoi* FE6 (1997) FE60 (2005)	Adriatic Sea (Mediterranean Sea)	Guillard’s F/2 medium. 19 °C, 100 μmol·m^−2^·s^−1^ light intensity and 12 h:12 h photoperiod.	Late stationary phase	[8]

**Table 3:** Active marine-derived compounds available on the market. The table reports the producing marine organisms, the active compounds, the target cancer cell lines, the active concentrations and references.

Marine Organism	Compound	Target	Active Concentration	Reference
*Ecteinascidia turbinata*	Ecteinascidin/ Trabectedin (alkaloid)	MFC7 A549	0.6 ng/mL (IC_70_) 5.6 ng/mL (IC_70_)	[38]
*Dolabella auricularia*/*Symploca sp.* VP642	Brentuximab vedotin (antibody-drug conjugate)	Non-Hodgkin’s lymphoma cells (Karpas 299)	2.5 ng/mL (IC_50_)	[39]
*Halichondria okadai*	Eribulin mesylate (macrolide)	DLD-1 LNCaP HL-60	6.934 ng/mL (IC_50_) 0.365 ng/mL (IC_50_) 0.657 ng/mL (IC_50_)	[40]
*Cryptotheca crypta*	Cytarabine (nucleoside)	Acute Myeloid Leukemia (AML) cells	272 ng/mL (IC_50_)	[41]

**Table 4:** Active marine-derived compounds in phase III clinical trials. The table reports the producing marine organisms, the active compounds, the target cancer cell lines, the active concentrations and references.

Marine Organism	Compound	Target	Active Concentration	Reference
*Aspergillus* sp. CNC139	Plinabulin (diketopiperazine)	Multiple myeloma cells (MM.1S, MM.1R, RPMI8226, and INA-6)	2.7 to 3.375 ng/mL (IC_50_)	[42]
*Aplidium albicans*	Plitidepsin (depsipeptide)	MCF-7	55.5 ng/mL (IC_50_)	[43]
*Halichondria okadai*	Lurbinectedin (alkaloid)	Ovarian cancer cells (RMG1, RMG2, KOC7C, HAC2, A2780, HeyA8 and SKOV-3)	0.78 to 2.34 ng/mL (IC_50_)	[44]

## References

[1] What Is Cancer. https://www.cancer.gov/about-cancer/understanding/what-is-cancer.

[2] European Cancer Observatory. http://eco.iarc.fr/.

[3] Dyshlovoy S.A., Honecker F. (2015). Marine compounds and cancer: Where do we stand?. Mar. Drugs.

[4] Jaspars M., De Pascale D., Andersen J.H., Reyes F., Crawford A.D., Ianora A. (2016). The marine biodiscovery pipeline and ocean medicines of tomorrow. J. Mar. Biol. Assoc. UK.

[5] Van Norman G.A. (2016). Drugs, Devices, and the FDA: Part 1: An Overview of Approval Processes for Drugs. JACC Basic Transl. Sci..

[6] Biopharmaceutical Research & Development. http://phrma-docs.phrma.org/sites/default/files/pdf/rd_brochure_022307.pdf.

[7] Moreno-Garrido I. (2008). Microalgae immobilization: Current techniques and uses. Bioresour. Technol..

[8] Lauritano C., Andersen J.H., Hansen E., Albrigtsen M., Escalera L., Esposito F., Helland K., Hanssen K.Ø., Romano G., Ianora A. (2016). Bioactivity screening of microalgae for antioxidant, anti-inflammatory, anticancer, anti-diabetes and antibacterial activities. Front. Mar. Sci..

[9] Romano G., Costantini M., Sansone C., Lauritano C., Ruocco N., Ianora A. (2017). Marine microorganisms as a promising and sustainable source of bioactive molecules. Mar. Environ. Res..

[10] De Morais M.G., Vaz B.D.S., De Morais E.G., Costa J.A.V. (2015). Biologically active metabolites synthesized by microalgae. Biomed. Res. Int..

[11] Miralto A., Barone G., Romano G., Poulet S.A., Ianora A., Russo G.L., Buttino I., Mazzarella G., Laabir M., Cabrini M. (1999). The insidious effect of diatoms on copepod reproduction. Nature.

[12] Sansone C., Braca A., Ercolesi E., Romano G., Palumbo A., Casotti R., Francone M., Ianora A. (2014). Diatom-derived polyunsaturated aldehydes activate cell death in human cancer cell lines but not normal cells. PLoS ONE.

[13] Kwang H.C., Song Y.I.K., Lee D.U. (2008). Antiproliferative effects of carotenoids extracted from *Chlorella ellipsoidea* and *Chlorella vulgaris* on human colon cancer cells. J. Agric. Food Chem..

[14] Kusaikin M.I., Ermakova S.P., Shevchenko N.M., Isakov V.V., Gorshkov A.G., Vereshchagin A.L., Grachev M.A., Zvyagintseva T.N. (2010). Structural characteristics and antitumor activity of a new chrysolaminaran from the diatom alga *Synedra acus*. Chem. Nat. Compd..

[15] Pasquet V., Morisset P., Ihammouine S., Chepied A., Aumailley L., Berard J.B., Serive B., Kaas R., Lanneluc I., Thiery V. (2011). Antiproliferative activity of violaxanthin isolated from bioguided fractionation of *Dunaliella tertiolecta* extracts. Mar. Drugs.

[16] Nappo M., Berkov S., Massucco C., Di Maria V., Bastida J., Codina C., Avila C., Messina P., Zupo V., Zupo S. (2012). Apoptotic activity of the marine diatom *Cocconeis scutellum* and eicosapentaenoic acid in BT20 cells. Pharm. Biol..

[17] Peng J., Yuan J.P., Wu C.F., Wang J.H. (2011). Fucoxanthin, a marine carotenoid present in brown seaweeds and diatoms: Metabolism and bioactivities relevant to human health. Mar. Drugs.

[18] Nigjeh S.E., Yusoff F., Banu N., Alitheen M., Rasoli M., Keong Y.S., Rahman A. (2013). Cytotoxic effect of ethanol extract of microalga, *Chaetoceros calcitrans*, and its mechanisms in inducing apoptosis in human breast cancer cell line. Biomed. Res. Int..

[19] Goh S.H., Alitheen N.B., Yusoff F.M., Yap S.K., Loh S.P. (2014). Crude ethyl acetate extract of marine microalga, *Chaetoceros calcitrans*, induces Apoptosis in MDA-MB-231 breast cancer cells. Pharmacogn. Mag..

[20] Samarakoon K.W., Ko J.Y., Shah M.M.R., Lee J.H., Kang M.C., O-Nam K., Lee J.B., Jeon Y.J. (2013). In vitro studies of anti-inflammatory and anticancer activities of organic solvent extracts from cultured marine microalgae. Algae.

[21] Shah M.R., Kalpa W.S., Ju-Young K., Lakmal H.H.C., Ji-Hyeok L., So-Jeong A., You-Jin J., Joon-Baek L. (2014). Potentiality of benthic dinoflagellate cultures and screening of their bioactivities in Jeju Island, Korea. Afr. J. Biotechnol..

[22] Kim Y.-S., Li X.-F., Kang K.-H., Ryu B., Kim S.-K. (2014). Stigmasterol isolated from marine microalgae *Navicula incerta* induces apoptosis in human hepatoma HepG2 cells. BMB Rep..

[23] Samarakoon K.W., Ko J.Y., Lee J.H., Kwon O.N., Kim S.W., Jeon Y.J. (2014). Apoptotic anticancer activity of a novel fatty alcohol ester isolated from cultured marine diatom, *Phaeodactylum tricornutum*. J. Funct. Foods.

[24] Andrianasolo E.H., Haramaty L., Vardi A., White E., Lutz R., Falkowski P. (2008). Apoptosis-inducing galactolipids from a cultured marine diatom, *Phaeodactylum tricornutum*. J. Nat. Prod..

[25] Somasekharan S.P., El-Naggar A., Sorensen P.H., Wang Y., Cheng H. (2016). An aqueous extract of marine microalgae exhibits antimetastatic activity through preferential killing of suspended cancer cells and anticolony forming activity. Evid. Based Complement. Altern. Med..

[26] Lin P.-Y., Tsai C.-T., Chuang W.-L., Chao Y.-H., Pan I.-H., Chen Y.-K., Lin C.-C., Wang B.-Y. (2017). *Chlorella sorokiniana* induces mitochondrial-mediated apoptosis in human non-small cell lung cancer cells and inhibits xenograft tumor growth in vivo. BMC Complement. Altern. Med..

[27] Ingebrigtsen R.A., Hansen E., Andersen J.H., Eilertsen H.C. (2016). Light and temperature effects on bioactivity in diatoms. J. Appl. Phycol..

[28] Ribalet F., Wichard T., Pohnert G., Ianora A., Miralto A., Casotti R. (2007). Age and nutrient limitation enhance polyunsaturated aldehyde production in marine diatoms. Phytochemistry.

[29] Dockery N., Higashida K., Verdes R.P., Mooneyham T.P. (2005). *Chlorella* Containing Nutritional Supplement Having Improved Digestability.

[30] Hildebrand M., Manandhar-Shrestha K., Abbriano R. (2017). Effects of chrysolaminarin synthase knockdown in the diatom *Thalassiosira pseudonana*: Implications of reduced carbohydrate storage relative to green algae. Algal Res..

[31] Chajès V., Sattler W., Stranzl A., Kostner G.M. (1995). Influence of n-3 fatty acids on the growth of human breast cancer cells in vitro: Relationship to peroxides and vitamin-E. Breast Cancer Res. Treat..

[32] Kong Z., Kao N., Hu J., Wu C. (2016). Fucoxanthin-rich brown algae extract decreases inflammation and attenuates colitis-associated colon cancer in mice. J. Food Nutr. Res..

[33] Kadekaru T., Toyama H., Yasumoto T. (2008). Safety evaluation of Fucoxanthin purified from *Undaria pinnatifida*. Nippon Shokuhin Kagaku Kogaku Kaishi.

[34] Ishikawa C., Tafuku S., Kadekaru T., Sawada S., Tomita M., Okudaira T., Nakazato T., Toda T., Uchihara J.N., Taira N. (2008). Antiadult T-cell leukemia effects of brown algae fucoxanthin and its deacetylated product, fucoxanthinol. Int. J. Cancer.

[35] Hosokawa M., Wanezaki S., Miyauchi K., Kurihara H., Kohno H., Kawabata J., Odashima S., Takahashi K. (1999). Apoptosis-inducing effect of fucoxanthin on human leukemia cell line HL-60. Food Sci. Technol. Res..

[36] Kotake-nara E., Kushiro M., Zhang H., Sugawara T., Miyashita K., Nagao A. (2001). Carotenoids affect proliferation of human prostate cancer cells. J. Nutr..

[37] Ryu B., Li Y., Qian Z.J., Kim M.M., Kim S.K. (2009). Differentiation of human osteosarcoma cells by isolated phlorotannins is subtly linked to COX-2, iNOS, MMPs, and MAPK signaling: Implication for chronic articular disease. Chem. Biol. Interact..

[38] Ghielmini M., Colli E., Erba E., Bergamaschi D., Pampallona S., Jimeno J., Faircloth G., Sessa C. (1998). In vitro schedule-dependency of myelotoxicity and cytotoxicity of Ecteinascidin 743 (ET-743). Ann. Oncol..

[39] Francisco J.A., Cerveny C.G., Meyer D.L., Mixan B.J., Klussman K., Chace D.F., Rejniak S.X., Gordon K.A., DeBlanc R., Toki B.E. (2003). cAC10-vcMMAE, an anti-CD30-monomethyl auristatin E conjugate with potent and selective antitumor activity. Blood.

[40] Towle M.J., Salvato K.A., Budrow J., Wels B.F., Kuznetsov G., Aalfs K.K., Welsh S., Zheng W., Seletsky B.M., Palme M.H. (2001). In vitro and in vivo anticancer activities of synthetic macrocyclic ketone analogues of halichondrin B. Cancer Res..

[41] Desai U., Shah K., Mirza S., Panchal D., Parikh S., Rawal R. (2015). Enhancement of the cytotoxic effects of Cytarabine in synergism with Hesperidine and Silibinin in Acute Myeloid Leukemia: An in-vitro approach. J. Cancer Res. Ther..

[42] Singh A.V., Bandi M., Raje N., Richardson P., Palladino M.A., Anderson K.C., Singh A.V., Bandi M., Raje N., Richardson P. (2011). A novel vascular disrupting agent plinabulin triggers JNK-mediated apoptosis and inhibits angiogenesis in multiple myeloma cells A novel vascular disrupting agent plinabulin triggers JNK-mediated apoptosis and inhibits angiogenesis in multiple myeloma cells. Blood.

[43] Gómez-Fabre P.M., De Pedro E., Medina M.A., Núñez De Castro I., Márquez J. (1997). Polyamine contents of human breast cancer cells treated with the cytotoxic agents chlorpheniramine and dehydrodidemnin B. Cancer Lett..

[44] Takahashi R., Mabuchi S., Kawano M., Sasano T., Matsumoto Y., Kuroda H., Kozasa K., Hashimoto K., Sawada K., Kimura T. (2016). Preclinical investigations of PM01183 (Lurbinectedin) as a single agent or in combination with other anticancer agents for clear cell carcinoma of the ovary. PLoS ONE.

[45] Monomethyl Auristatin E (MMAE). https://adcreview.com/adc-university/adcs-101/cytotoxic-agents/monomethyl-auristatin-e-mmae/.

[46] Cuevas C., Francesch A. (2009). Development of Yondelis^®^ (trabectedin, ET-743). A semisynthetic process solves the supply problem. Nat. Prod. Rep..

[47] Amaro H., Guedes A., Malcata F. (2011). Antimicrobial activities of microalgae: An invited review. Sci. Microb. Pathog. Commun. Curr. Res. Technol. Adv..

[48] Paul C., Reunamo A., Lindehoff E., Bergkvist J., Mausz M.A., Larsson H., Richter H., Wängberg S.Å., Leskinen P., Bam̊stedt U. (2012). Diatom derived polyunsaturated aldehydes do not structure the planktonic microbial community in a mesocosm study. Mar. Drugs.

[49] Swanson D., Block R., Mousa S.A. (2012). Omega-3 Fatty Acids EPA and DHA: Health benefits throughout life. Adv. Nutr..

[50] Chew K.W., Yap J.Y., Show P.L., Suan N.H., Juan J.C., Ling T.C., Lee D.J., Chang J.S. (2017). Microalgae biorefinery: High value products perspectives. Bioresour. Technol..

[51] Zhang H., Tang Y., Zhang Y., Zhang S., Qu J., Wang X., Kong R., Han C., Liu Z. (2015). Fucoxanthin: A promising medicinal and nutritional ingredient. Evid. Based Complement. Altern. Med..

[52] Luo X., Su P., Zhang W. (2015). Advances in microalgae-derived phytosterols for functional food and pharmaceutical applications. Mar. Drugs.

[53] Panda S., Jafri M., Kar A., Meheta B.K. (2009). Thyroid inhibitory, antiperoxidative and hypoglycemic effects of stigmasterol isolated from Butea monosperma. Fitoterapia.

[54] Saeidnia S. (2014). The Story of Beta-sitosterol—A Review. Eur. J. Med. Plants.

